# CheXaid: deep learning assistance for physician diagnosis of tuberculosis using chest x-rays in patients with HIV

**DOI:** 10.1038/s41746-020-00322-2

**Published:** 2020-09-09

**Authors:** Pranav Rajpurkar, Chloe O’Connell, Amit Schechter, Nishit Asnani, Jason Li, Amirhossein Kiani, Robyn L. Ball, Marc Mendelson, Gary Maartens, Daniël J. van Hoving, Rulan Griesel, Andrew Y. Ng, Tom H. Boyles, Matthew P. Lungren

**Affiliations:** 1 Stanford University Department of Computer Science, Stanford, CA USA; 2 Massachusetts General Hospital Department of Anesthesia, Boston, MA USA; 3 Stanford University AIMI Center, Stanford, CA USA; 4 Department of Medicine, University of Cape Town, Cape Town, South Africa

**Keywords:** Diagnosis, Machine learning

## Abstract

Tuberculosis (TB) is the leading cause of preventable death in HIV-positive patients, and yet often remains undiagnosed and untreated. Chest x-ray is often used to assist in diagnosis, yet this presents additional challenges due to atypical radiographic presentation and radiologist shortages in regions where co-infection is most common. We developed a deep learning algorithm to diagnose TB using clinical information and chest x-ray images from 677 HIV-positive patients with suspected TB from two hospitals in South Africa. We then sought to determine whether the algorithm could assist clinicians in the diagnosis of TB in HIV-positive patients as a web-based diagnostic assistant. Use of the algorithm resulted in a modest but statistically significant improvement in clinician accuracy (*p* = 0.002), increasing the mean clinician accuracy from 0.60 (95% CI 0.57, 0.63) without assistance to 0.65 (95% CI 0.60, 0.70) with assistance. However, the accuracy of assisted clinicians was significantly lower (*p* < 0.001) than that of the stand-alone algorithm, which had an accuracy of 0.79 (95% CI 0.77, 0.82) on the same unseen test cases. These results suggest that deep learning assistance may improve clinician accuracy in TB diagnosis using chest x-rays, which would be valuable in settings with a high burden of HIV/TB co-infection. Moreover, the high accuracy of the stand-alone algorithm suggests a potential value particularly in settings with a scarcity of radiological expertise.

## Introduction

Tuberculosis (TB) was responsible for the deaths of an estimated 300,000 patients living with HIV in 2017, making it the leading cause of preventable HIV-related mortality^1^. More than 14 million individuals worldwide are estimated to be dually infected with HIV and *Mycobacterium tuberculosis*, yet TB remains undetected at the time of death in ~46% of patients with HIV^2,3^. Since TB is both treatable and often fatal in patients with HIV, improving its diagnosis is of the utmost importance^4^.

Diagnosis of TB is a significant challenge in patients with HIV due to nonspecific clinical presentations that can mimic other respiratory diseases common in this population, as well as frequently atypical (or even normal) chest radiographs^5,6^. In addition, patients with HIV are often sputum scarce, presenting a challenge for microbiological diagnosis with culture or Xpert MTB/RIF^7,8^. In this clinically challenging setting, chest radiographs can provide a valuable adjunct diagnostic tool, particularly for seriously ill patients requiring immediate treatment^1,9^. Current WHO TB diagnostic algorithms recommend the use of chest x-ray as part of the diagnostic work-up in both ambulatory and seriously ill HIV-positive patients when initial microbiological assays are either negative or unavailable^1^. However, chest x-ray interpretation in patients with advanced HIV is challenging due to the presence of atypical findings, and because access to experienced radiologists is often limited in countries where HIV and TB are most prevalent^8,9^. This is particularly true in sub-Saharan Africa, where ~86% of co-infection deaths occur^10^.

Computer-aided diagnostic tools, particularly those utilizing deep learning, have recently emerged as a potential solution to the gap in TB diagnostic expertise^11–21^. While such approaches have shown promise in assisted medical imaging interpretation, application of deep learning to assist clinicians in their diagnosis of TB in patients with HIV remains unexplored^22–27^. The development of an accurate deep learning algorithm to help clinicians diagnose active TB in these patients without sputum, or in whom sputum tests are negative, has the potential to provide physicians with an easily accessible and immediately applicable diagnostic support tool for this vulnerable population, and improve access to radiology expertise on a task commonly performed by generalist physicians.

In this study, we developed a TB diagnostic algorithm and assistant to help clinicians in the diagnosis of TB using chest x-rays in patients co-infected with HIV. The algorithm was developed to leverage both the patient’s radiograph and relevant clinical information together, and the assistant consisted of a web interface capable of incorporating the algorithm’s predictions and explanation into a simulated diagnostic workflow. We measured the effect of this assistance on the diagnostic performance of clinicians and compared it to the stand-alone model performance.

## Results

**Fig. 1:**
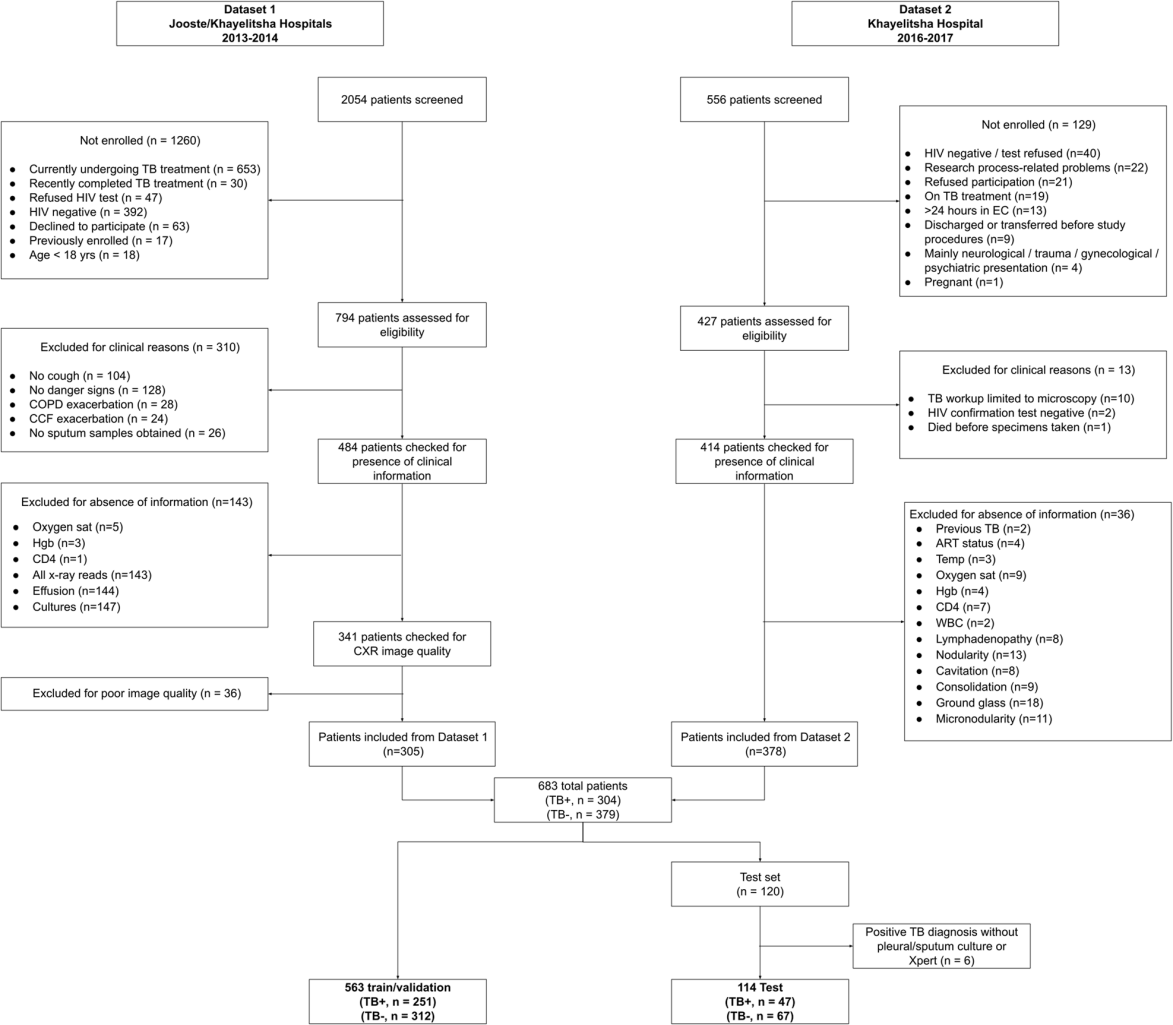
Flow diagram of patients. Patient screening, recruitment, and inclusion for each dataset are summarized.

Two different datasets consisting of patients in South Africa with possible HIV/TB co-infection were combined and randomly split into a training set (*n* = 563, prevalence = 44.6%) used to train and select algorithms, and a held out test set (*n* = 114, prevalence = 41.2%) used to evaluate the final algorithms. There was no overlap in radiographs between the two datasets. Patient screening, recruitment, and inclusion for each dataset are summarized in Fig. 1, and detailed in the Supplementary Note 1. The sizes of training and testing splits along with the TB diagnostic prevalence in each dataset are detailed in Supplementary Table 1. Table 1 contains demographic and clinical summary statistics for each dataset.

**Table 1:** Cohort demographic and clinical characteristics by dataset and TB diagnosis.

	Dataset 1 Jooste/Khayelitsha (*n* = 305)		Dataset 2 Khayelitsha (*n* = 378)		All participants (*n* = 677)
	TB (*n* = 146)	No TB (*n* = 159)	TB (*n* = 158)	No TB (*n* = 220)	
Variable	Mean (SD)	Mean (SD)	Mean (SD)	Mean (SD)	Mean (SD)
Age (years)	35.9 (9.5)	38.6 (10.5)	36.5 (9.4)	37.9 (9.7)	37.3 (9.8)
Temperature (°C)	38.0 (1.3)	37.5 (1.3)	37.1 (1.2)	37.0 (1.2)	37.3 (1.3)
Oxygen saturation (%)	96 (5)	94 (7)	96 (4)	95 (5)	95 (6)
Hemoglobin (mg/dL)	8.8 (2.3)	10.7 (2.3)	9.0 (2.4)	10.3 (2.7)	9.8 (2.6)
WBC count (1000/µL)	8.7 (5.1)	11.7 (6.7)	9.7 (9.7)	11.8 (11.4)	10.7 (9)
CD4 count (cells/mm^3^)	127 (117)	203 (200)	116 (151)	203 (274)	167 (208)

	*n* (%)	*n* (%)	*n* (%)	*n* (%)	*n* (%)
Sex: female	96 (66%)	109 (69%)	95 (60%)	238 (58%)	425 (63%)
Current cough	146 (100%)	159 (100%)	136 (85%)	186 (85%)	623 (92%)
Previous TB	96 (66%)	128 (81%)	67 (42%)	133 (60%)	422 (62%)
Currently on ART	49 (34%)	64 (40%)	57 (36%)	125 (57%)	292 (43%)

### Physicians with and without assistance

We developed a deep learning algorithm, called CheXaid, to diagnose active pulmonary TB from both chest x-rays and clinical covariates. Using CheXaid, we performed a diagnostic accuracy study comparing physicians with and without algorithm assistance at the task of diagnosing active pulmonary TB for HIV positive patients. A clinician operating without algorithm assistance only had access to the web interface that showed the original x-ray image and the full set of clinical variables for every patient. A clinician operating with algorithm assistance also had access to the algorithm’s prediction (on a scale from very unlikely to very likely) and visual explanation of the prediction. A total of 13 physicians were recruited from email mailing lists for physicians in South Africa. All had completed training, with anywhere from 6 months to 25 years of experience diagnosing TB in patients with HIV in South Africa. Subspecialties represented included hospitalists, general practitioners, family medicine specialists, or casualty officers.

Assistance increased physicians’ accuracy according to the mixed effects model likelihood ratio test (chi-square = 9.64, unadjusted *p* value = 0.002), a difference that reached statistical significance. Physicians’ mean accuracy (correct divided by total cases) was 0.60 (95% CI 0.57, 0.63) without the algorithm and 0.65 (95% CI 0.60, 0.70) with assistance. Sensitivity was 0.70 (95% CI 0.64, 0.77) without assistance and 0.73 (95% CI 0.66, 0.80) with assistance; specificity was 0.52 (95% CI 0.45, 0.59) without assistance and 0.61 (95% CI 0.52, 0.70) with assistance.

### Assisted physicians vs. stand-alone algorithm

**Fig. 2:**
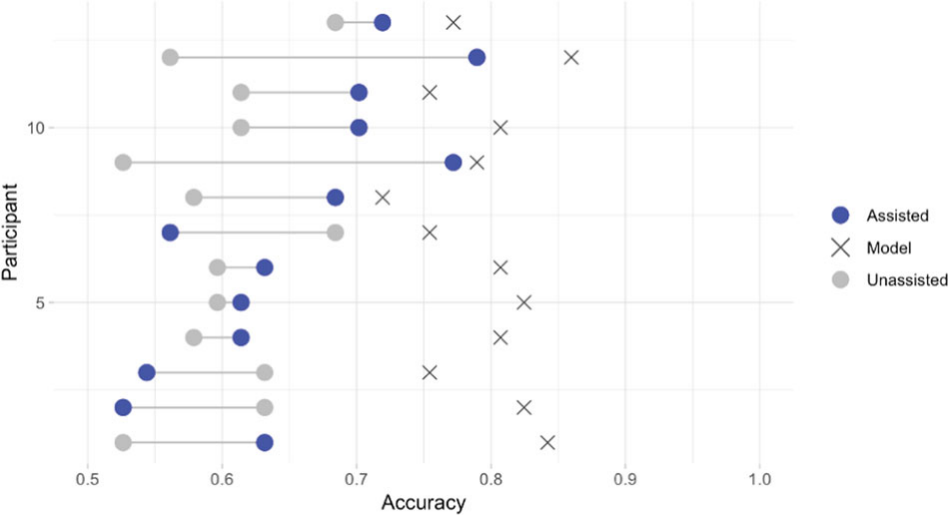
Diagnostic accuracy of the assisted physicians, stand-alone algorithm, and unassisted physicians. Each cross represents the stand-alone algorithm’s performance on test data that was assigned as assisted cases for the correspondent physician.

The mean accuracy of the assisted physicians was significantly lower than the stand-alone algorithm (chi-square = 66.6, unadjusted *p* value < 0.0001). The stand-alone algorithm achieved a mean accuracy of 0.79 (95% CI 0.77, 0.82) evaluated on the cases the physicians viewed with algorithm assistance, while physicians achieved a mean assisted accuracy of 0.65 (95% CI 0.60, 0.70) on these same cases. The stand-alone algorithm had a sensitivity of 0.67 (95% CI 0.62, 0.73) and specificity of 0.87 (95% CI 0.85, 0.90). Mean accuracy, sensitivity, specificity, PPV, and NPV without and with assistance are detailed in Supplementary Table 2. Figure 2 (in addition to Supplementary Table 3) details the accuracy of each of the individual physicians with and without assistance.

### Algorithm performance under different training strategies

Evaluated on the full test set, the algorithm achieved an accuracy of 0.78 (95% CI 0.70, 0.85) and AUC of 0.83 (95% CI 0.75, 0.91) evaluated on all of the independent test set. Trained without clinical covariates, the algorithm achieved an accuracy of 0.61 (95% CI 0.51, 0.69) and AUC of 0.57 (95% CI 0.46, 0.68). Without pre-training on a very large dataset of chest x-rays, but still pre-trained with ImageNet, the algorithm achieved an accuracy of 0.64 (95% CI 0.55, 0.72), and AUC of 0.71 (95% CI 0.62, 0.81). The algorithm performance under different training strategies is detailed in Table 2.

**Table 2:** Algorithm performance under four training strategies.

Strategy	Accuracy (95% CI)	AUC (95% CI)
Default (w/ Clinical Variables, w/ CheXpert Pretraining, w/ Multi-Label Loss)	0.78 (0.70, 0.85)	0.83 (0.75, 0.91)
Default w/o Clinical Variables	0.61 (0.51, 0.69)	0.57 (0.46, 0.68)
Default w/o CheXpert Pre-training	0.64 (0.55, 0.72)	0.71 (0.62, 0.81)
Default trained on dataset one, validated on dataset two	0.67 (0.62, 0.72)	0.71 (0.65, 0.76)
Default trained on dataset two, validated on dataset one	0.60 (0.55, 0.66)	0.70 (0.64, 0.76)

The default uses clinical variables, pretraining on CheXpert, and the multi-label loss.

## Discussion

The purpose of this study was to develop and investigate a deep learning algorithm to assist physicians in the diagnosis of TB in patients with HIV. Our approach can incorporate both chest radiograph image data as well as relevant structured clinical information to make a diagnostic decision; furthermore, the physician-facing interface is capable of presenting the model’s predictions into a convenient diagnostic workflow to assist in clinical interpretation.

There is a significant opportunity for application of deep learning assistants like CheXaid for TB diagnosis in HIV-positive populations. Currently, there are a variety of diagnostic approaches to TB in this challenging population; when sputum can be produced, Xpert MTB/RIF is the recommended test in these patients as it can provide same-day high-sensitivity correlation with smear-positive TB and rifampin resistance^28^. Despite its well-documented reliability, Xpert MTB/RIF nonetheless remains limited by high cost and limited availability, which has led to extensive underutilization particularly in the most vulnerable HIV-positive populations. In these settings, as few as 4% of co-infected patients are diagnosed using the recommended Xpert MTB/RIF test, and less reliable and time-intensive diagnostic approaches—such as smear microscopy—are largely relied upon for diagnosis^29,30^. Given that underutilization of Xpert MTB/RIF for TB diagnosis is common in HIV-positive populations, CheXaid assisted chest radiographic interpretation may serve as an important aid to clinicians, particularly if smear microscopy is inconclusive.

We found that CheXaid improved clinician accuracy (60 vs. 65%, chi-square = 9.64, *p* = 0.002), a difference that was statistically significant though modest in size and therefore of unclear clinical significance. While the observed 5% increase in accuracy may not be of large clinical significance, these results suggest an opportunity to leverage scalable inexpensive portable applications to aid clinicians as part of a more comprehensive toolset for TB diagnosis. Furthermore, our finding that the mean accuracy of assisted clinicians was significantly lower (60 vs. 79%, chi-square = 66.6, unadjusted *p* value < 0.001) than that of the stand-alone algorithm on the same unseen test cases suggests a possible role for use of the algorithm underlying CheXaid as a stand-alone tool when access to experts is limited. Possible explanations of our finding that the stand-alone algorithm performed better than clinicians who access to its output (79 vs. 65%) include some degree of mistrust of the algorithm’s output or overconfidence in a clinician’s own diagnosis when submitting a final answer. Alternatively, the representation of the algorithm’s diagnostic probability as a category (one of five, from unlikely to very likely) rather than a percent may have introduced additional uncertainty in cases in which the probability was estimated near 50% (i.e., in the “possible” category). Future research should examine the extent to which the performance of physicians can be improved with additional clinical data or different interpretation methodologies and interfaces for the algorithm’s decision-making process. Ultimately, prospective evaluation is necessary to determine whether similar algorithms can be beneficial in a clinical setting.

There has been some prior work in applications of deep learning to classify TB and/or identify lesions in chest radiographs, with some reporting considerable success (some with AUCs as high as 0.98–0.99) mainly with datasets composed of healthy or asymptomatic screening populations or heterogeneous datasets with variable ground truth^11,12^. But in HIV positive populations, diagnosis using chest radiographs is much more challenging even for trained radiologists, with sensitivities and specificities as low as 0.68 and 0.53, respectively^31^. In this challenging setting, clinicians must also consider clinical data when interpreting chest radiographs for possible TB infection, as these variables may influence radiological presentation and disease detection^32,33^. Prior work has attempted other strategies for integration of demographic data in deep learning medical imaging applications, such as using gender and age demonstrating only limited improvement in model performance in a “late fusion” schema^19,33^. Incorporation of demographic data has also been explored as a way to triage the use of Xpert testing in resource-constrained settings^14^. By providing structured clinical data in our modeling along with a clinician-centered interface, our model was able to achieve an accuracy 0.79 (95% CI 0.77, 0.82). Without clinical information, the algorithm’s accuracy was only 0.61 (95% CI 0.51, 0.69), which is similar to that of unassisted clinicians (mean accuracy 0.60, 95% CI 0.57, 0.63), indicating that clinical information had a large influence on the algorithm’s predicted diagnosis.

This study has several important limitations. First, while the gold standard diagnostic tool for active pulmonary TB is generally considered to be culture, we also included patients whose sputum or pleural fluid tested positive using the Xpert MTB/RIF test. This diagnostic method has been shown to have a high sensitivity and specificity for TB detection, even in patients with HIV, yet the discrepancy should be noted^34^. Furthermore, although the high specificity of Xpert MTB/RIF (92–100%) makes false positives unlikely, the two culture/Xpert MTB/RIF requirement does not ensure that both tests were successful and there remains a possibility of false negatives in the test set^35^. For patients in the training set, we did not require that the positive culture or Xpert MTB/RIF test come specifically from pleural fluid or sputum. This was to avoid excluding patients unnecessarily from the training set, while allowing us to maintain a more stringent case definition limiting false positives in the test set. However, despite the limitations of available data, we believe that the use of microbiological confirmation rather than consensus opinion is an overall strength of the study. Second, while chest x-ray is a valuable tool for TB diagnosis in certain settings (i.e., urgent diagnosis or ruling out active disease prior to treating latent infection), it is not as sensitive or specific as culture or Xpert MTB/RIF testing, and does not provide information on drug resistance. As a result, the WHO recommends the Xpert MTB/RIF (and now Xpert MTB/RIF Ultra) as a first diagnostic step for patients with suspected HIV/TB co-infection when immediate referral to a higher level of care is not possible^36^. With regard to the data used for testing and training, the fact that patients were recruited from a common hospital for both datasets raises the possibility that there were patients in the test set that were also used for training. However, for a patient to be included in both datasets, the corresponding x-ray and clinical information would correspond to time points separated by over a year, and therefore knowing a patient’s previous TB status would not necessarily have given the model any additional information about their current status. Third, while we describe the stand-alone CheXaid model performed consistently better than clinicians aided by the model in our study, it is important to note when interpreting these data that the task for this analysis was narrowly defined as simply diagnosis of active TB, while clinical decisions (i.e., whether or not to prescribe treatment) are much more complex. Furthermore, the use of clinical trial data resulted in the exclusion of certain important patient populations due to presence of diagnoses that were excluded from the original trials. For example, patients experiencing a current exacerbation of COPD or heart failure from one of the studies, although in clinical practice, these conditions (whether concurrent with TB or as an alternative diagnosis) may make the diagnosis of TB using chest radiographs more challenging. Additional important pulmonary diagnoses, while not excluded, were not considered by CheXaid (i.e., *Streptococcus pneumoniae, Pneumocystis jirovecii)* and thus future implementation efforts based on these data would always include clinician input and consideration of these diagnoses in practice. Additional patients were also excluded due to missing data or poor quality x-ray images. In the case of missing data, multiple imputation was not performed in this case because of the relatively large number of patients who were missing multiple variables (i.e., no findings reported on chest x-ray). While the exclusion of patients due to “poor quality x-ray images” was performed in this case by a board-certified radiologist, the criteria for inclusion comprised of minimal quality standards (i.e., containing the full lung field, or cell phone image of the x-ray not in focus) that we believe could be performed by an x-ray technician in resource-scarce settings. Fourth, the resolution of the images presented both to the algorithm and to physicians was not optimized for diagnostic quality, and future studies should explore the implementation of similar algorithms employing full resolution images for both training and evaluation. Finally, clinicians were not given access to the full spectrum of data that is available when making this diagnosis in practice, including physical exam, a detailed history, and other laboratory values. While this was necessitated by the use of clinical trial data, further work should include prospective studies that allow clinicians to evaluate patients with and without the algorithm in real time, allowing them to collect and consider this information in their ultimate diagnosis and allow for a more accurate comparison to true clinical practice.

In conclusion, our study found that a deep learning model was effective in predicting TB from chest x-rays and clinical information of HIV-positive patients, and its assistance can significantly improve clinician performance in TB diagnosis, but not to the level of diagnostic performance of the stand-alone algorithm. If more clinical data can be incorporated, and the model can be validated prospectively in clinical settings with larger and more diverse groups of patients, a tool like this could be valuable in settings with high prevalence of HIV/TB co-infection, where radiological expertise is scarce and access to low-cost solutions is needed.

## Methods

### Dataset

Two different datasets were used in this study, each consisting of patients in South Africa with possible HIV/TB co-infection. The first dataset was collected as part of a prospective cohort study of adult inpatients with HIV, cough of any duration and at least one WHO danger sign from two secondary level hospitals serving communities with a high burden of HIV and TB in Cape Town, South Africa: GF Jooste Hospital (November 2011–February 2013), and Khayelitsha Hospital (March 2013–October 2014)^37^. The second dataset was collected as part of a cross-sectional diagnostic study of HIV-positive patients with at least one TB symptom admitted to the emergency center of Khayelitsha Hospital from 2016 to 2017^38^.

### Reference standard

Positive cases were defined as having a positive culture or Xpert MTB/RIF test from any anatomical site. This was due to the high pretest probability of active pulmonary TB in this population (including disseminated TB also including the lungs), and because not all sputum samples collected resulted in a conclusive positive or negative test result^7,8^. Because the outcome of interest for this study was a diagnosis of active pulmonary TB, in the test set, positive cases were required to have at least one positive culture or Xpert MTB/RIF result from sputum or pleural fluid. Each patient was required to have had at least 2 sputum cultures collected (with sputum induction in the cases in which sputum could not be expectorated) in both training and test datasets.

### AI algorithm development

**Fig. 3:**
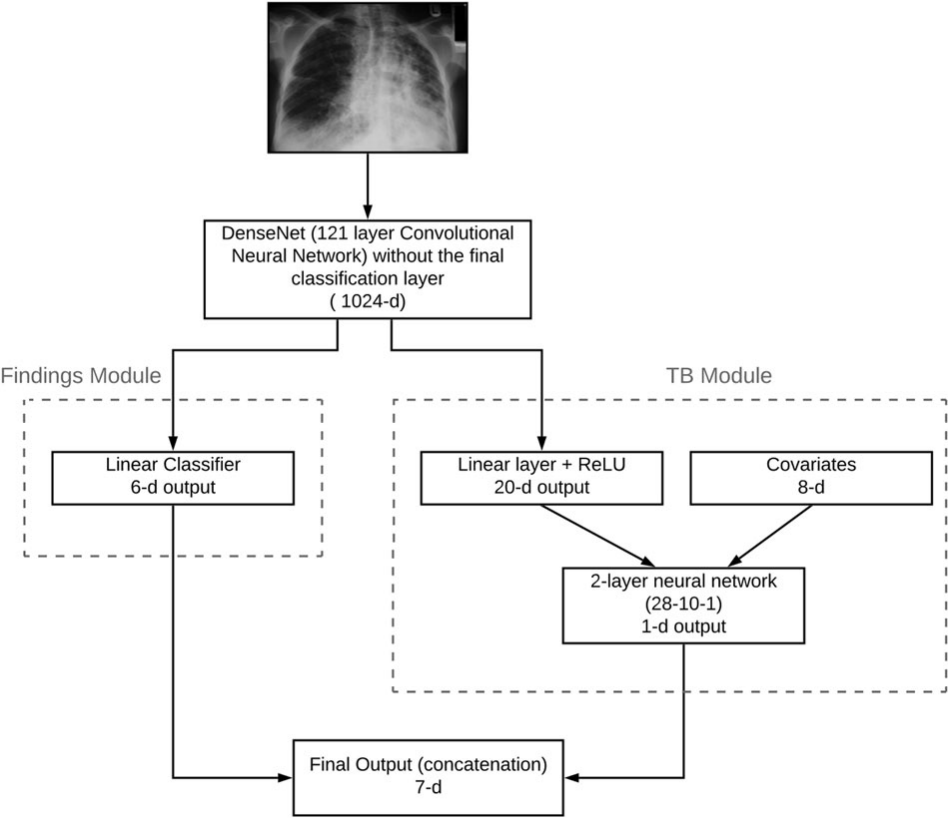
Diagram of the deep learning algorithm architecture. The architecture takes both the x-ray image and 8 clinical covariates as input and predicts TB and 6 clinical findings.

CheXaid was developed to diagnose active pulmonary TB from both chest x-rays and clinical covariates. The algorithm development is detailed in the Supplementary Note 2. The clinical covariates inputted to the algorithm were age, oxygen saturation, hemoglobin, CD4 count, white blood cell count, temperature, current antiretroviral therapy status, and the patient’s prior history of TB; covariates were selected based on availability in both datasets. The algorithm was trained not only to predict the probability of TB, but also the presence of six x-ray findings to further supervise the algorithm towards learning relevant x-ray features for TB diagnosis: micronodularity, nodularity, pleural effusion, cavitation, and ground-glass. The algorithm was pre-trained on a very large dataset of chest x-rays (CheXpert), which included 224,316 chest radiographs of 65,240 patients, with labels for 14 radiological observations, but not for TB, before fine-tuning on the training set. Figure 3 shows a diagrammatic representation of the deep learning algorithm architecture.

### AI diagnostic assistant development

We developed a web-based TB diagnostic assistant for clinicians that integrated the algorithm’s predictions and the following “explanatory” information. First, the algorithm’s estimated likelihood of the patient having active pulmonary TB was presented in five categories were based on these probabilities (of the patient having active pulmonary TB): very unlikely (0.0–0.2), unlikely (0.2–0.4), possible (0.4–0.6), likely (0.6–0.8), and very likely (0.8–1.0). In addition, the assistance interface also incorporated an explanation of prediction that highlighted the regions of the x-ray which were most consistent with TB according to the algorithm.

### Experimental setup

**Fig. 4:**
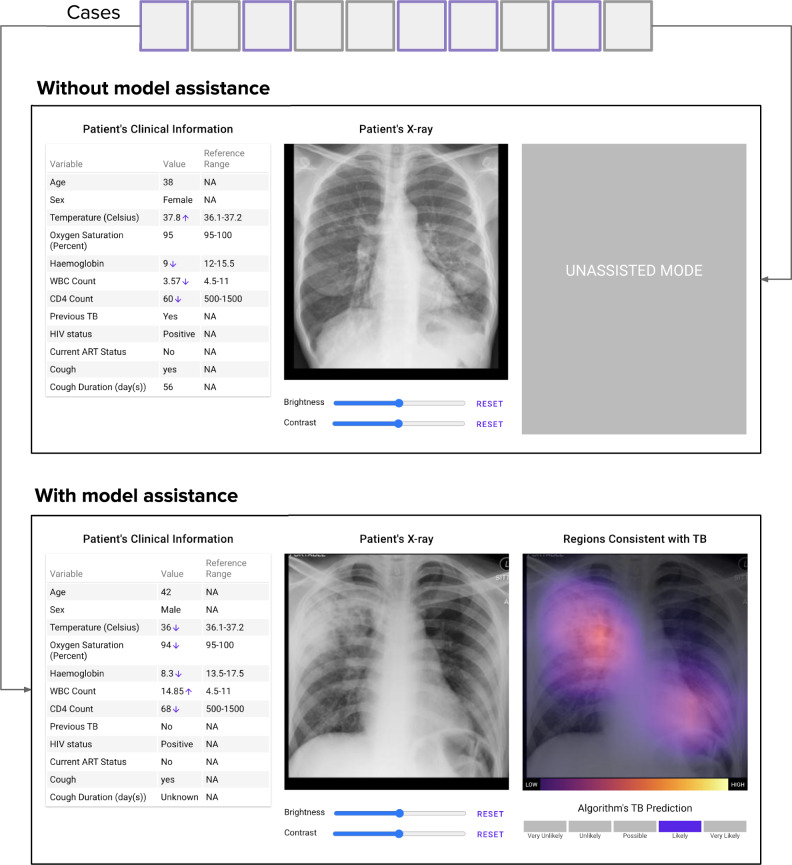
Experimental setup. Test cases were randomly assigned to be diagnosed assisted/unassisted by the clinicians. Each clinician analyzed half the cases without algorithm assistance and half of them with algorithm assistance. The web interface for unassisted and assisted viewing is illustrated. Upon examining each case, the clinician made a prediction for the likelihood of a positive TB diagnosis.

To compare performance metrics of clinicians with and without algorithm assistance, each of the 13 clinicians participating in the study diagnosed the test set (of 114 cases) designed as a within-subjects, intermodal, multi-reader study. The clinicians were blinded to the original reports, clinical histories (beyond the clinical covariates provided), and follow-up imaging examinations. Clinicians interpreted half of the study cases with assistance from the algorithm and half of them without (Fig. 4). Cases were randomly assigned to the “assisted” vs. “unassisted” conditions on a per-subject basis. Case order was also randomized across clinicians in order to avoid confounding by reader fatigue. In order to familiarize the clinicians with the diagnostic assistant and help them overcome the learning curve, each experiment began with a training session, in which they reviewed up to 104 training cases (distinct from the testing cases) assisted by the algorithm’s output. During this training session, the true diagnosis was presented immediately after the clinician submitted their diagnosis. Stanford University institutional review board approved this study, and ethics review and Institutional Review Board (IRB) review and approval was obtained from the University of Cape Town in South Africa. Informed consent was obtained from all human participants.

### Statistical analyses

The diagnostic accuracy of the physicians with and without assistance was assessed on the test set using a logistic mixed effects model. Physicians and cases were included as random effects and an indicator for whether the case was read with assistance or not was treated as a fixed effect. In order to evaluate the effect of algorithm assistance, the full model was compared to a restricted model without the indicator for assistance with the likelihood-ratio test; significance was assessed at the 0.01 level.

Similarly, the diagnostic accuracy of the assisted physicians was compared to the accuracy of the algorithm’s diagnostic predictions without clinician supervision (stand-alone algorithm) using logistic mixed-effects models, except in this analysis only the cases assigned to the assisted condition were used per participant, and the fixed effect of interest was an indicator for whether the case was assessed by the assisted physician or the stand-alone algorithm. The binarized version of the algorithm’s prediction was determined using a threshold of 0.5. All models were generated using the “lme4” package in R^39,40^.

95% *t*-score confidence intervals were computed for the accuracy, sensitivity, and specificity of the physicians with and without assistance as a group (*n* = 13). A similar analysis was performed for the stand-alone algorithm, with summary statistics computed per-participant using only the cases assigned to the assisted condition. 95% Wilson score confidence intervals were used to assess the variability in the estimates for accuracy, sensitivity, and specificity per-physician for the unassisted physician, assisted physician, and stand-alone algorithm.

A sensitivity analysis was performed to determine the performance of the algorithm’s diagnostic performance over all examples in the test set, under different training strategies. First, we compared the diagnostic accuracy and receiver operating characteristic area under the curve (AUC) of the final algorithm to one that was not trained on clinical variables. Second, we compared the final algorithm, pre-trained on CheXpert, to one that was pre-trained on ImageNet. Variability in estimates were assessed using the DeLong’s method for the AUC, and the Wilson’s score method for accuracy.

### Reporting summary

Further information on research design is available in the Nature Research Reporting Summary linked to this article.

## Supplementary information

Supplementary Information

Reporting Summary

## Data Availability

The raw clinical patient data that support the findings of this study are available from the original authors of the observational studies (G.M. for first dataset^37^, and D.J.H. for second dataset^38^) for which they were collected but restrictions apply to the availability of these data, which were used under license for the current study, and so are not publicly available. Diagnostic accuracy data for algorithm, assisted and unassisted physicians are available in aggregated form in Supplementary Table 3 and the full dataset is available from the corresponding author on reasonable request.

## Code availability

Code used in this study can be found in https://github.com/stanfordmlgroup/CheXaid.
