# Supplementary material for: CheXaid: deep learning assistance for physician diagnosis of tuberculosis using chest x-rays in patients with HIV
Source: NPJ Digit Med. 2020 Sep 9;3:115. doi: 10.1038/s41746-020-00322-2 (PMC7481246; doi:10.1038/s41746-020-00322-2)
Supplement: Supplementary file 1 — Supplementary Information [file 41746_2020_322_MOESM1_ESM.pdf]

Supplementary Note 1

Dataset One Recruitment and Inclusion

The first dataset was collected with a prospective cohort study of inpatient adults with HIV and suspected TB. Chest x-rays and clinical information were collected from consecutively recruited inpatient adults with HIV, any duration of cough, and WHO danger signs. Inclusion criteria were: HIV-infected, ≥18 years, within the first 24 hours of hospital admission, cough of any duration, and ≥1 WHO danger signs (either of: respiratory rate >30 breaths/minute, heart rate >120 beats/minute, temperature >39°C, and being unable to walk unaided). Exclusion criteria were: anti-tuberculosis therapy that is current or completed in the previous month or defaulted within the past 6 months, exacerbation of cardiac failure or chronic obstructive pulmonary disease, and inability to produce a spontaneous or induced sputum sample. Participants received a standardized work-up for their respiratory illness including a chest radiograph and CD4 count unless a CD4 count was available within six months prior to admission. Sputum samples were taken from all participants: one sample for Gram stain, culture and sensitivity; and two samples for smear examination with auramine staining for acid-fast bacilli (AFB) and mycobacterial culture. On one sample sent for mycobacterial culture the Xpert MTB/RIF assay was performed. Sputum was induced using an ultrasonic nebulizer and hypertonic saline (5%) when participants were unable to produce sputum spontaneously. A mycobacterial blood culture (BacT/Alert® specimen bottle) was performed on all patients. Following clinical trial completion, patients with missing data were excluded, most frequently because their chest x-rays had not been read by a radiologist (n = 143). Multiple imputation was not performed due to the fact that the majority of these patients were missing multiple datapoints (i.e. presence of nodularity, effusion, lymphadenopathy) and therefore any imputation scheme would have performed poorly. In addition, a basic quality check was performed on the x-ray images to ensure that x-rays met basic quality standards (i.e. included the entirety of the lung parenchyma and
x-rays met basic quality standards (i.e. contained both inferior angles of the diaphragm and entirety of the lung parenchyma).

Dataset Two Recruitment and Inclusion

The second dataset was collected as part of a cross-sectional diagnostic study of HIV-infected patients with at least one TB symptom (current cough, fever, night sweats or weight loss) admitted to the emergency center of Khayelitsha Hospital from 2016-2017. Inclusion criteria were: ≥ 18 years of age, HIV-positive, and currently experiencing at least one TB symptom. Inclusion criteria were: patients on anti-TB treatment (currently or within the past 3 months), patients admitted longer than 24 hours to the emergency center, informed consent not obtained, main clinical presenting feature of meningitis syndrome or new focal neurology, trauma, gynecological or psychiatric-related presentation, or pregnant. All patients underwent systematic testing for TB including CD4 count, chest x-ray, point-of-care ultrasound, sputum and urine Xpert MTB/RIF assays (Cepheid, Sunnyvale, CA, USA) and culture, and urine LAM assays performed in the emergency center and independent laboratory (Alere Determine™ TB LAM Ag, Waltham, MA, USA).

Patient Characteristics In Both Datasets

In dataset one, patients with TB had slightly lower hemoglobin (mean hemoglobin 8.8 vs. 10.7), white blood cell (WBC) counts (mean 8.7 vs. 11.7), and CD4 counts (mean 127 vs. 203). In dataset 2, patients with TB also had lower WBC counts (9.7 vs. 11.8), but discrepancy in hemoglobin was slightly less pronounced (mean hemoglobin 9.0 vs. 10.3). Again, patients with TB had lower CD4 counts on average (mean 116 vs. 203). Both datasets were predominantly female (66% and 69% with and without TB in dataset one, and 60% and 58% with and without TB in dataset 2). All of the patients in dataset one reported cough (as this was one of the inclusion criteria), while only 85% of the patients in dataset two reported cough.

Supplementary Note 2
Algorithm architecture

Neural networks are complex functions with many parameters structured as a hierarchy of layers to model different levels of abstraction. A convolutional neural network, a particular type of neural network is specially designed to handle image data: inspired by the organization of neurons in a human visual cortex, a convolutional neural network takes advantage of a parameter sharing receptive field to learn local features of an image and abstractions of these local features.

The neural network consists of two components. First, a 121-layer DenseNet (pretrained on CheXpert as discussed above) architecture was used to extract image features as a 1024-dimensional vector. In the DenseNet architecture, each layer is directly connected to every other layer within a block; for each layer, the feature maps of all preceding layers are used as inputs, and its own feature maps are passed on to all following layers as inputs. The network then forks into two modules, one for TB diagnosis using the image features and the clinical covariates, and the other for predicting the occurrence of six clinical findings that were diagnosed by radiologists and should be inferable just from the X-ray image. The TB module first uses a linear layer to learn 20 image features from the original 1024 dimensions, and then combines them with the 8 covariates, feeding the resulting 28-dimensional patient representation into a two layer neural network to predict TB. The findings module is a linear multi-label classifier on top of the 1024-dimensional image representation with 6 output units.

Focal Loss:
We optimized the algorithm's parameters with a focal loss applied to TB classification, as well as classification of additional X-ray findings. The loss for TB is upweighted by a factor of 6 so that it contributes as much as all the additional findings together. The focal loss is a modification to the standard cross entropy loss that includes an additional factor, $-(1-p_y)_c$, where y is the target label and c > 0 is a hyperparameter. The overall loss for a single example is $focal\_loss(p_y) = -(1-p_y)_c * \log(p_y)$. The additional factor downweights the loss for examples that are easy to classify (i.e. when the probability of correct class is close to 1), and thus gives greater importance to examples that are challenging to classify.

Preprocessing
Images were cropped to exclude regions outside of the lungs using human annotations of the lung regions. After cropping, the shorter dimension was padded so that the resulting image was a square. Images were then downsampled to an input resolution of 320x320. Finally, the pixel values were normalized based on the per-channel mean and standard deviation that had been precomputed on the training set. All numerical clinical covariates (i.e. CD4 count, WBC count, etc.) were also normalized using the mean and standard deviation of the training set.

Algorithm Hyperparameters
The Adam optimizer with default parameters for beta1 (0.9) and beta2 (0.999) was used. The batch size was set to 16 and the initial learning rate was set to 1e-4. The learning rate was halved every 500 iterations. We also added L2 regularization to the algorithm parameters with a weight decay of 1e-4. Additionally, we applied random affine transformations (translation, rotation, and scaling) for data augmentation during training.

Visual Interpretation of the algorithm
Class activation maps (CAMs) were used to highlight regions that had the greatest influence on the algorithm's decision. CAMs were generated by taking the weighted average across the algorithm's final convolutional feature maps, with weights based on global average pooling for each map. This averaged map was then scaled by the outputted probability so that more confident predictions appear brighter. Finally, the map was upsampled to the input image resolution and overlaid onto the input image.

Ensembling

We performed 5-fold cross validation in training our algorithm. On each fold, we evaluated performance on the validation split every 512 iterations and chose the best algorithm based on the highest validation AUROC. We used the best algorithm trained on each fold in an ensemble algorithm. During inference, each of the five algorithms in the ensemble produces a probability of TB and these probabilities are then averaged to get a final, ensemble probability.

For CAMs, we generated a CAM from each algorithm in the ensemble and then averaged them to get a single, ensemble CAM. The scaling is also based on the final, ensemble probability.

Other details

Clinicians diagnosed the test cases on their own time. In addition, clinicians were given information on the duration of a patient's cough, although this information was not used to train the algorithm given its presence in only one of the datasets.

Supplementary Tables

Supplementary Table 1: Training and Testing splits, along with the TB diagnosis.

	Training<br>n (%)	Test<br>n (%)
Number of Positive Cases	251 (45%)	47 (42%)
Number of Negative Cases	312 (55%)	67 (58%)
Total Cases	563	114

Supplementary Table 2: Diagnostic Performance of the physicians only, model only, and physician and model as a group, along with 95% confidence intervals. Accuracy, sensitivity, specificity, and their confidence intervals are shown.

	Accuracy (95% CI)	Sensitivity (95% CI)	Specificity (95% CI)	PPV (95% CI)	NPV (95%CI)
Clinicians Assisted	0.65 (0.60, 0.70)	0.73 (0.66, 0.80)	0.61 (0.52, 0.70)	0.55 (0.48, 0.62)	0.76 (0.74, 0.83)
Clinicians Unassisted	0.60 (0.57, 0.63)	0.70 (0.64, 0.77)	0.52 (0.45, 0.59)	0.54 (0.51, 0.57)	0.70 (0.65, 0.74)
Stand-alone Algorithm	0.79 (0.77, 0.82)	0.67 (0.62, 0.72)	0.87 (0.85, 0.90)	0.76 (0.72, 0.81)	0.81 (0.78, 0.84)

Supplementary Table 3: Diagnostic Performance of the individual physicians only, model only, and physician and model, along with 95% confidence intervals. "Algorithm X only" reports the performance of the algorithm when tested on the subset of cases that physician X diagnosed in assisted mode.

	Accuracy (95% CI)	Sensitivity (95% CI)	Specificity (95% CI)	PPV (95% CI)	NPV (95% CI)
Physician 1 only	0.61 (0.48, 0.73)	0.71 (0.51, 0.85)	0.55 (0.38, 0.7)	0.53 (0.36, 0.69)	0.72 (0.52, 0.86)
Physician 1 with Algorithm	0.7 (0.57, 0.8)	0.65 (0.45, 0.81)	0.74 (0.57, 0.85)	0.62 (0.43, 0.79)	0.76 (0.59, 0.87)
Algorithm 1 only	0.75 (0.63, 0.85)	0.67 (0.47, 0.82)	0.82 (0.66, 0.91)	0.75 (0.58, 0.87)	0.76 (0.57, 0.89)
Physician 2 only	0.56 (0.43, 0.68)	0.52 (0.33, 0.71)	0.59 (0.42, 0.74)	0.46 (0.29, 0.65)	0.65 (0.47, 0.79)
Physician 2 with Algorithm	0.79 (0.67, 0.88)	0.67 (0.47, 0.82)	0.88 (0.73, 0.95)	0.8 (0.58, 0.92)	0.78 (0.63, 0.89)
Algorithm 2 only	0.7 (0.57, 0.8)	0.61 (0.41, 0.78)	0.76 (0.6, 0.88)	0.62 (0.43, 0.78)	0.77 (0.6, 0.89)
Physician 3 only	0.58 (0.45, 0.7)	0.85 (0.68, 0.94)	0.33 (0.19, 0.51)	0.53 (0.39, 0.67)	0.71 (0.45, 0.88)
Physician 3 with Algorithm	0.68 (0.56, 0.79)	0.8 (0.58, 0.92)	0.62 (0.46, 0.76)	0.53 (0.36, 0.7)	0.85 (0.68, 0.94)
Algorithm 3 only	0.84 (0.73, 0.91)	0.78 (0.59, 0.89)	0.9 (0.74, 0.97)	0.86 (0.73, 0.93)	0.79 (0.52, 0.92)
Physician 4 only	0.58 (0.45, 0.7)	0.56 (0.37, 0.72)	0.6 (0.42, 0.75)	0.56 (0.37, 0.72)	0.6 (0.42, 0.75)
Physician 4 with Algorithm	0.61 (0.48, 0.73)	0.7 (0.48, 0.85)	0.57 (0.41, 0.71)	0.47 (0.3, 0.64)	0.78 (0.59, 0.89)
Algorithm 4 only	0.75 (0.63, 0.85)	0.67 (0.48, 0.81)	0.83 (0.66, 0.93)	0.67 (0.48, 0.81)	0.83 (0.66, 0.93)
Physician 5 only	0.68 (0.56, 0.79)	0.6 (0.41, 0.77)	0.75 (0.58, 0.87)	0.65 (0.45, 0.81)	0.71 (0.54, 0.83)
Physician 5 with Algorithm	0.72 (0.59, 0.82)	0.91 (0.72, 0.97)	0.6 (0.44, 0.74)	0.59 (0.42, 0.74)	0.91 (0.73, 0.98)
Algorithm 5 only	0.79 (0.67, 0.88)	0.68 (0.48, 0.83)	0.88 (0.72, 0.95)	0.78 (0.58, 0.9)	0.79 (0.63, 0.9)
Physician 6 only	0.53 (0.4, 0.65)	0.72 (0.52, 0.86)	0.38 (0.23, 0.55)	0.47 (0.32, 0.63)	0.63 (0.41, 0.81)

Physician 6 with Algorithm	0.77 (0.65, 0.86)	0.73 (0.52, 0.87)	0.8 (0.64, 0.9)	0.7 (0.49, 0.84)	0.82 (0.66, 0.92)
Algorithm 6 only	0.77 (0.65, 0.86)	0.68 (0.48, 0.83)	0.84 (0.68, 0.93)	0.76 (0.61, 0.87)	0.79 (0.57, 0.91)
Physician 7 only	0.6 (0.47, 0.71)	0.75 (0.55, 0.88)	0.48 (0.33, 0.65)	0.51 (0.36, 0.67)	0.73 (0.52, 0.87)
Physician 7 with Algorithm	0.63 (0.5, 0.74)	0.83 (0.63, 0.93)	0.5 (0.34, 0.66)	0.53 (0.37, 0.68)	0.81 (0.6, 0.92)
Algorithm 7 only	0.75 (0.63, 0.85)	0.62 (0.43, 0.79)	0.85 (0.69, 0.93)	0.8 (0.64, 0.9)	0.68 (0.47, 0.84)
Physician 8 only	0.63 (0.5, 0.74)	0.64 (0.45, 0.8)	0.62 (0.45, 0.77)	0.57 (0.39, 0.73)	0.69 (0.51, 0.83)
Physician 8 with Algorithm	0.54 (0.42, 0.67)	0.5 (0.31, 0.69)	0.57 (0.41, 0.72)	0.42 (0.26, 0.61)	0.65 (0.47, 0.79)
Algorithm 8 only	0.81 (0.69, 0.89)	0.72 (0.52, 0.86)	0.88 (0.72, 0.95)	0.82 (0.64, 0.92)	0.79 (0.62, 0.9)
Physician 9 only	0.53 (0.4, 0.65)	0.73 (0.54, 0.86)	0.35 (0.21, 0.53)	0.49 (0.34, 0.64)	0.61 (0.39, 0.8)
Physician 9 with Algorithm	0.63 (0.5, 0.74)	0.76 (0.55, 0.89)	0.56 (0.4, 0.7)	0.5 (0.34, 0.66)	0.8 (0.61, 0.91)
Algorithm 9 only	0.72 (0.59, 0.82)	0.54 (0.35, 0.71)	0.87 (0.71, 0.95)	0.77 (0.62, 0.87)	0.61 (0.39, 0.8)
Physician 10 only	0.6 (0.47, 0.71)	0.62 (0.43, 0.78)	0.58 (0.41, 0.74)	0.55 (0.38, 0.72)	0.64 (0.46, 0.79)
Physician 10 with Algorithm	0.61 (0.48, 0.73)	0.67 (0.45, 0.83)	0.58 (0.42, 0.73)	0.48 (0.31, 0.66)	0.75 (0.57, 0.87)
Algorithm 10 only	0.74 (0.61, 0.83)	0.58 (0.39, 0.74)	0.87 (0.71, 0.95)	0.69 (0.51, 0.83)	0.79 (0.6, 0.9)
Physician 11 only	0.63 (0.5, 0.74)	0.88 (0.7, 0.96)	0.44 (0.28, 0.61)	0.55 (0.4, 0.69)	0.82 (0.59, 0.94)
Physician 11 with Algorithm	0.53 (0.4, 0.65)	0.91 (0.72, 0.97)	0.29 (0.16, 0.45)	0.44 (0.31, 0.59)	0.83 (0.55, 0.95)
Algorithm 11 only	0.74 (0.61, 0.83)	0.52 (0.33, 0.7)	0.91 (0.76, 0.97)	0.62 (0.47, 0.76)	1 (0.82, 1)
Physician 12 only	0.61 (0.48, 0.73)	0.74 (0.54, 0.87)	0.53 (0.37, 0.69)	0.52 (0.35, 0.67)	0.75 (0.55, 0.88)

Physician 12 with Algorithm	0.7 (0.57, 0.8)	0.67 (0.47, 0.82)	0.73 (0.56, 0.85)	0.64 (0.45, 0.8)	0.75 (0.58, 0.87)
Algorithm 12 only	0.81 (0.69, 0.89)	0.65 (0.45, 0.81)	0.91 (0.77, 0.97)	0.76 (0.59, 0.87)	0.88 (0.69, 0.96)
Physician 13 only	0.68 (0.56, 0.79)	0.84 (0.65, 0.94)	0.56 (0.39, 0.72)	0.6 (0.44, 0.74)	0.82 (0.61, 0.93)
Physician 13 with Algorithm	0.56 (0.43, 0.68)	0.68 (0.47, 0.84)	0.49 (0.33, 0.64)	0.45 (0.3, 0.62)	0.71 (0.51, 0.85)
Algorithm 13 only	0.81 (0.69, 0.89)	0.72 (0.52, 0.86)	0.88 (0.72, 0.95)	0.77 (0.61, 0.88)	0.86 (0.67, 0.95)
