# Supplementary material for: CheXaid: deep learning assistance for physician diagnosis of tuberculosis using chest x-rays in patients with HIV
Source: NPJ Digit Med. 2020 Sep 9;3:115. doi: 10.1038/s41746-020-00322-2 (PMC7481246; doi:10.1038/s41746-020-00322-2)
Supplement: Supplementary file 2 — Reporting Summary [file 41746_2020_322_MOESM2_ESM.pdf]

nature research

Corresponding author(s): Pranav Rajpurkar

Last updated by author(s): Jun 13, 2020

Reporting Summary

Nature Research wishes to improve the reproducibility of the work that we publish. This form provides structure for consistency and transparency in reporting. For further information on Nature Research policies, see our Editorial Policies and the Editorial Policy Checklist.

Statistics

For all statistical analyses, confirm that the following items are present in the figure legend, table legend, main text, or Methods section.

n/a	Confirmed	
☐	☒	The exact sample size (n) for each experimental group/condition, given as a discrete number and unit of measurement
☐	☒	A statement on whether measurements were taken from distinct samples or whether the same sample was measured repeatedly
☐	☒	The statistical test(s) used AND whether they are one- or two-sided<br>*Only common tests should be described solely by name; describe more complex techniques in the Methods section.*
☐	☒	A description of all covariates tested
☐	☒	A description of any assumptions or corrections, such as tests of normality and adjustment for multiple comparisons
☐	☒	A full description of the statistical parameters including central tendency (e.g. means) or other basic estimates (e.g. regression coefficient) AND variation (e.g. standard deviation) or associated estimates of uncertainty (e.g. confidence intervals)
☐	☒	For null hypothesis testing, the test statistic (e.g. F, t, r) with confidence intervals, effect sizes, degrees of freedom and P value noted<br>*Give P values as exact values whenever suitable.*
☒	☐	For Bayesian analysis, information on the choice of priors and Markov chain Monte Carlo settings
☒	☐	For hierarchical and complex designs, identification of the appropriate level for tests and full reporting of outcomes
☐	☒	Estimates of effect sizes (e.g. Cohen's d, Pearson's r), indicating how they were calculated

Our web collection on statistics for biologists contains articles on many of the points above.

Software and code

Policy information about availability of computer code

Data collection	The custom web-based interface used to collect physician interpretations was written in HTML/Javascript.
Data analysis	Code used for this project (in Python v3.6) can be found at https://github.com/stanfordmlgroup/chexaid

For manuscripts utilizing custom algorithms or software that are central to the research but not yet described in published literature, software must be made available to editors and reviewers. We strongly encourage code deposition in a community repository (e.g. GitHub). See the Nature Research guidelines for submitting code & software for further information.

Data

Policy information about availability of data

All manuscripts must include a data availability statement. This statement should provide the following information, where applicable:

- Accession codes, unique identifiers, or web links for publicly available datasets
- A list of figures that have associated raw data
- A description of any restrictions on data availability

The raw clinical patient data that support the findings of this study are available from the original authors of the clinical trials for which they were collected but restrictions apply to the availability of these data, which were used under license for the current study, and so are not publicly available. Diagnostic accuracy data for algorithm, assisted and unassisted physicians is available in aggregated form in Supplementary Table 3 and the full dataset is available from the corresponding author on reasonable request.

Field-specific reporting

Please select the one below that is the best fit for your research. If you are not sure, read the appropriate sections before making your selection.

☒ Life sciences ☐ Behavioural & social sciences ☐ Ecological, evolutionary & environmental sciences

For a reference copy of the document with all sections, see nature.com/documents/nr-reporting-summary-flat.pdf

Life sciences study design

All studies must disclose on these points even when the disclosure is negative.

Sample size

The sample size was determined by the number of available cases that meet the exclusion criteria described in the manuscript.

Data exclusions

The first dataset was collected with a prospective cohort study of inpatient adults with HIV and suspected TB. Inclusion criteria were: HIV-infected, ≥18 years, within the first 24 hours of hospital admission, cough of any duration, and ≥1 WHO danger signs (either of: respiratory rate >30 breaths/minute, heart rate >120 beats/minute, temperature >39°C, and being unable to walk unaided). Exclusion criteria were: anti-tuberculosis therapy that is current or completed in the previous month or defaulted within the past 6 months, exacerbation of cardiac failure or chronic obstructive pulmonary disease, and inability to produce a spontaneous or induced sputum sample. Following clinical trial completion, patients with missing data were excluded, most frequently because their chest x-rays had not been read by a radiologist. The second dataset was collected as part of a cross-sectional diagnostic study of HIV-infected patients with at least one TB symptom (current cough, fever, night sweats or weight loss) admitted to the emergency center of Khayelitsha Hospital from 2016-2017. Inclusion criteria were: ≥ 18 years of age, HIV-positive, and currently experiencing at least one TB symptom. Inclusion criteria were: patients on anti-TB treatment (currently or within the past 3 months), patients admitted longer than 24 hours to the emergency center, informed consent not obtained, main clinical presenting feature of meningitis syndrome or new focal neurology, trauma, gynecological or psychiatric-related presentation, or pregnant.

For AI diagnostic assistance study, there were no data exclusions.

Replication

The performance of the deep learning model was replicated by running the model on a held-out internal validation set, as well as an external test set.

Randomization

Randomization was used to split data to training, validation, and independent test sets.

For AI diagnostic assistance study, cases were randomly assigned to the "assisted" vs. "unassisted" conditions on a per-subject basis. Case order was also randomized across clinicians in order to avoid confounding by reader fatigue.

Blinding

All clinicians in this experiment were blinded to group allocation. Clinicians were blinded to the original reports, clinical histories (beyond the clinical covariates provided) and follow-up imaging examinations.

Reporting for specific materials, systems and methods

We require information from authors about some types of materials, experimental systems and methods used in many studies. Here, indicate whether each material, system or method listed is relevant to your study. If you are not sure if a list item applies to your research, read the appropriate section before selecting a response.

Materials & experimental systems

n/a	Involved in the study	
☒	☐	Antibodies
☒	☐	Eukaryotic cell lines
☒	☐	Palaeontology and archaeology
☒	☐	Animals and other organisms
☐	☒	Human research participants
☒	☐	Clinical data
☒	☐	Dual use research of concern

Methods

n/a	Involved in the study	
☒	☐	ChIP-seq
☒	☐	Flow cytometry
☒	☐	MRI-based neuroimaging

Human research participants

Policy information about studies involving human research participants

Population characteristics

A total of 13 physicians participated in the AI Assisted Diagnosis study. All had completed training, with anywhere from 6 months to 25 years of experience diagnosing TB in patients with HIV in South Africa. Subspecialties represented included hospitalists, general practitioners, family medicine specialists, or casualty officers.

Recruitment

All 13 physicians were recruited from email mailing lists for physicians in South Africa.

Ethics oversight	Stanford University, ethics review and Institutional Review Board (IRB) review and approval from the University of Cape Town in South Africa

Note that full information on the approval of the study protocol must also be provided in the manuscript.
